# Retraction: Activated K-ras and INK4a/Arf Deficiency Cooperate During the Development of Pancreatic Cancer by Activation of Notch and NF-κB Signaling Pathways

**DOI:** 10.1371/journal.pone.0205289

**Published:** 2018-10-02

**Authors:** The PLOS ONE Editors

After publication of this article [1], concerns were raised that lanes 1–12 of the Bcl-2 blot in Fig 3A are similar to the Notch-4 blot shown in Fig 1D, though the image appears to have been adjusted vertically and horizontally in one of the panels. The authors published a Correction [2] to address the duplication by providing an updated version of Fig 3A in which the Bcl-2 panel was replaced; raw blots supporting the new Bcl-2 data and the Notch-4 data were included in Supporting Information with the Correction.

It was subsequently raised that based on the Supporting Information file, the Bcl-2 data appear to have been stretched vertically in preparing the updated figure panel [2].

It was further noted that the IKKβ and Cyclin D1 panels appear similar in both the original and corrected versions of Fig 3A [1, 2], though one image appears stretched vertically relative to the other.

Wayne State University investigated the concerns raised about the Bcl-2/Notch-4 duplication and found evidence of data falsification. The investigating committee did not find files or documentation underlying the updated Bcl-2 blot included in the Correction or documentary support to verify that the Notch-4 and Bcl-2 data represented the indicated experiments.

In light of these concerns, and in line with the institution’s recommendation, the *PLOS ONE* Editors retract this article.

ASA, BB, RMM, LM, and MK agreed with the retraction. ZW, SB, AA, YL, JRG, DK, SA, JG and FHS did not respond.
